# No correlation exists between coracoid tunnel widening and loss of reduction after arthroscopic acromioclavicular fixation using tightrope system

**DOI:** 10.1007/s00167-023-07329-8

**Published:** 2023-02-20

**Authors:** Ahmed Samir Elkalyoby, Mohamed Refaat Waly, Begad Hesham Mostafa Zaky Abdelrazek, Ahmed Rizk Mohamed, Khaled Shohayeb, Ahmed Fouad Seifeldin

**Affiliations:** Trauma and Orthopedics Faculty of Medicine, Cairo University, Mathaf El-Manial Street, Manial Cairo, 11553 Egypt

**Keywords:** Acute acromioclavicular joint injury, TightRope, Coracoid tunnel widening, Loss of reduction

## Abstract

**Purpose:**

To detect the widening of the tunnel of the coracoid process after arthroscopic fixation of acute acromioclavicular joint (ACJ) dislocation using the TightRope system and its correlation with loss of reduction and functional scores.

**Methods:**

From 2016 to 2018, a prospective study was performed on twenty-three patients with acute grade III–V ACJ dislocation. Arthroscopic TightRope repair of the ACJ was performed. Coracoid tunnel widening was measured by CT, and the coracoclavicular distance was measured on the radiographs immediately postoperatively and at 12 months. The Constant Shoulder Score, Oxford Shoulder Score, Nottingham Clavicle Score and Visual analog scale were used as outcome measures at 12 months.

**Results:**

The coracoid tunnel diameter to horizontal coracoid diameter ratio increased from 22.8 ± 3.7% immediately postoperatively to 38.5 ± 5.5% at 12 months (*p* < 0.001). The coracoclavicular (CC) distance showed an increase from a mean of 10.8 ± 1.7 mm to a mean of 11.8 ± 2.5 at 12 months (*p* < 0.001). There was no correlation between the increase in the coracoclavicular distance and the patients’ functional clinical scores or coracoid tunnel widening.

**Conclusion:**

Coracoid tunnel widening and radiological loss of reduction occur after arthroscopic fixation of acute ACJ dislocation with the TightRope system. However, they do not correlate with each other or with the functional scores of the patient.

**Level of evidence:**

Level IV.

## Introduction

Injuries of the ACJ are among the most common injuries of the shoulder girdle, with an estimated incidence of 1.8 per 1000 per year [7]. The Rockwood classification is the most widely used system for classifying the severity of such injuries and deciding which treatment option should be used [25]. Grade IV–VI injuries require surgical intervention; however, the management of grade III injuries is debatable. While many surgeons advocate surgical treatment, others argue that with nonoperative management, patients do equally well [18]. However, the surgical treatment of ACJ injuries is still controversial due to lack of general agreement on the optimal technique [12].

The aim of treatment is to achieve anatomical reduction and stable fixation with motion preservation in addition to regaining the preinjury activity and functional state [8]. Arthroscopic techniques for ACJ injuries have been gaining popularity using smaller wounds to minimize the hospital stay, postoperative pain, and adhesions, which allows for quicker recovery and associated intra-articular injuries that can be diagnosed and managed [2].

The concept of arthroscopic-assisted fixation of acute ACJ injury is to use suspensory devices to maintain the coracoclavicular distance and facilitate healing of the CC and AC ligaments [21, 26]. These suspensory devices require the drilling of tunnels into the clavicle and the coracoid, which yields complications, such as fracture of the distal clavicle and coracoid and tunnel widening with biomechanical consequences [10, 22]. Also, loss of reduction is a commonly found complication with an incidence ranging from 12 to 80% after arthroscopic techniques [9, 11, 21, 29]. The clinical effect of tunnel widening on loss of reduction has been recently studied [4, 24, 30]. A clinical study on the effect of tunnel widening on the clavicle showed a significant increase in the tunnel diameter in the early postoperative period, which increases the risk of fracture [30]. However, coracoid tunnel widening and its clinical impact have not yet been studied.

The purpose of this study was to establish whether coracoid tunnel widening dilatation occurs with ACJ stabilization using the TightRope system (Arthrex, Naples, FL, USA) and to study whether there is any correlation between this widening and loss of reduction and whether it is clinically significant. The hypothesis was that significant coracoid tunnel widening would occur postoperatively and is a direct cause of loss of reduction.

## Materials and methods

From 2016 to 2018, a prospective study was performed at our institute. Institutional Review Board (IRB) approval was obtained from the faculty of medicine of Cairo University; number I-170518. Twenty-three patients with acute (≤ 3 weeks after injury) Rockwood grade III–V ACJ dislocation were included in the study. Patients with associated coracoid fracture, associated rotator cuff tears or glenohumeral arthritis were excluded. The mean age was 37.5 ± 10.4 years. Out of 23 patients, 21 were males (91.3%), and 2 were females (8.7%). Eleven patients (47.8%) were office-based employees, while twelve patients (52.2%) were manual workers. Six patients (26.1%) had type III ACJ dislocations, 4 patients (17.4%) had type IV dislocations, and 13 (56.5%) patients had type V dislocations. The right side was injured in 14 patients (60.9%), while the left side was involved in 9 patients (39.1%). Eighteen patients (78.3%) were right-hand dominant, while five (21.7%) patients were left-hand dominant. Three patients had associated injuries. Two patients had medial subluxation of the biceps tendon for which biceps tenotomy was performed, and one patient suffered a grade I superior labrum anterior to posterior (SLAP) lesion managed by arthroscopic debridement. All patients were evaluated clinically and radiologically using radiographs (AP, axillary lateral and Zanca views) and computed tomography (CT) scans to exclude coracoid fracture. Informed consent was obtained from all participants included in the study.

### Surgical technique

The arthroscopic procedure was performed in a beach-chair position under general anesthesia. After ensuring joint reducibility, a 1 cm vertical incision was made over the lateral end of the clavicle 3 cm medial to the AC joint, and then the deltotrapezial fascia was incised horizontally. Reduction was achieved and then maintained by temporary k-wire under C-arm visualization. Shoulder arthroscopy was then commenced through the standard posterior visualizing portal using a 30° arthroscope. Diagnostic shoulder arthroscopy was performed to exclude associated pathologies. The rotator interval was identified, and an anterolateral working portal was established just above the subscapularis by an outside-in technique preserving the middle glenohumeral ligament (MGHL) and medial biceps pulley. The accessory anterolateral (AAL) portal was established superior and lateral to the previous portal. Then, a radiofrequency probe was used to expose the base of the coracoid. The arthroscope is then switched to the AAL portal.

A specific C-guide for ACJ (Arthrex, Naples) was placed via the anterolateral portal under the base of the coracoid. Care was exercised to ensure that the guide was centered under the coracoid to avoid eccentric drilling or notching. The drill sleeve of the guide was placed over the lateral clavicle approximately 3 cm medial to the AC joint. A guide pin was introduced through the clavicle under arthroscopic visualization until it appeared on the coracoid undersurface. A cannulated drill-bit was used to drill a ***single*** 4-mm tunnel. The guide pin was removed, leaving the drill bit in place. A flexible looped Nitinol passing wire was passed down the drill bit and retrieved via the anterolateral portal. It was used to shuttle the fiber-wire of the tight rope, bringing the button to rest on the undersurface of the coracoid. The final reduction was confirmed by C-arm followed by securing the washer to the clavicle by tying the fiber-wire free ends together. Closure of the deltotrapezial fascia, subcutaneous tissue and skin was performed in separate layers.

The arm was immobilized in a broad arm sling allowing only passive range of motion in the supine position for six weeks. From 6 weeks postoperatively onward, active-assisted followed by active range-of-motion (ROM) exercises were allowed. This was followed by a gradual muscle strengthening and coordination program.

**Fig. 1:**
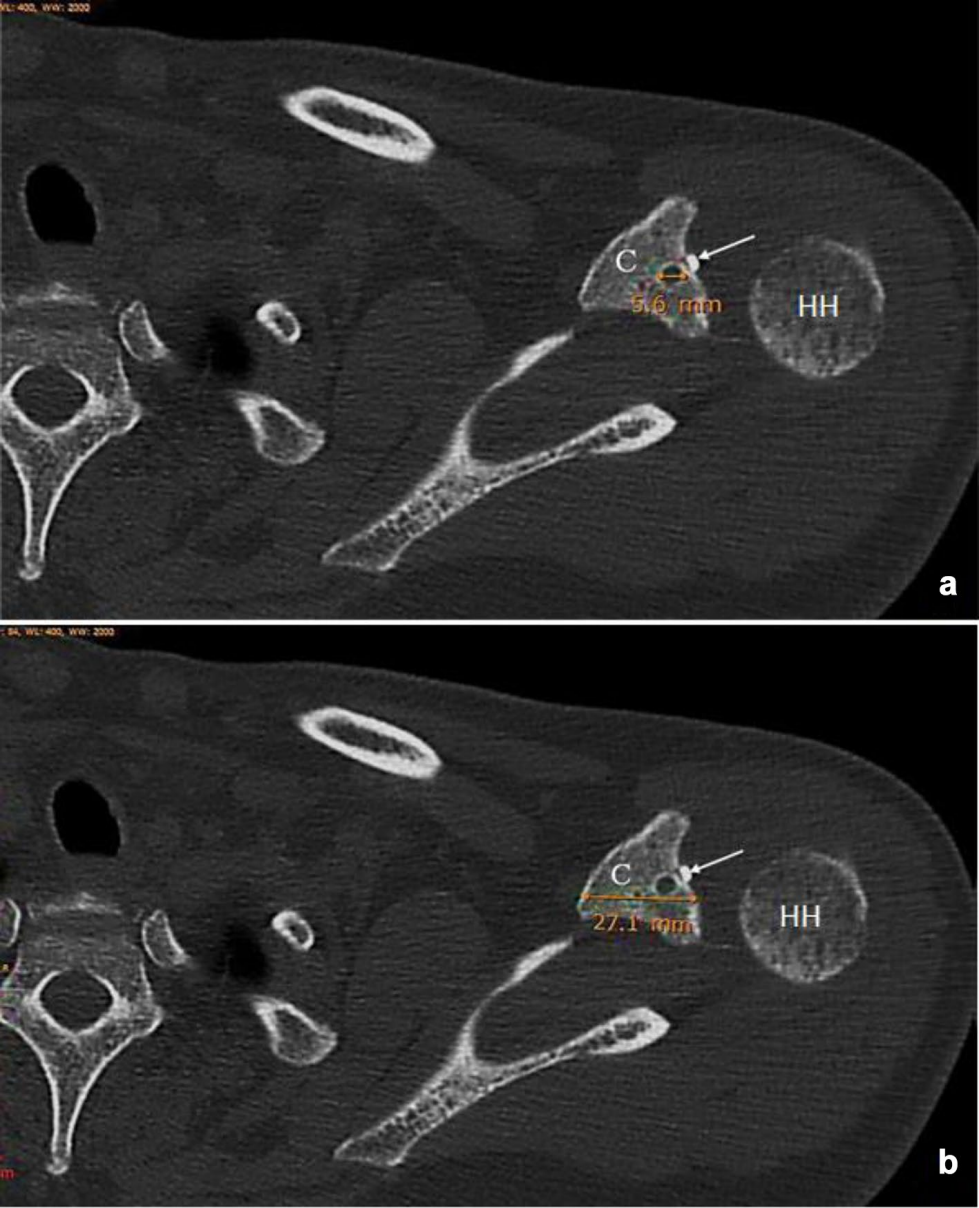
One-year postoperative CT scan axial cuts measuring **a** coracoid tunnel diameter at its widest width of 5.6 mm (orange line) and **b** coracoid horizontal diameter at its widest width of 27.1 mm (orange line). The ratio between both diameters was then calculated; Key: *C* coracoid, *HH* humeral head, white arrow for the endoutton

Immediate postoperative radiographs (Zanca view) were performed at 6 and 12 weeks and one year. The CC distance from the lowest point on the upper surface of the coracoid to the lower margin of the clavicle at a 90-degree angle was measured immediately postoperatively and at 12 months. Any loss of reduction was recorded, while radiological failure was defined as a ≥ 50% increase in the CC distance [8]. Computed tomography was performed immediately postoperatively and after one year to assess coracoid tunnel widening using RadiAnt DICOM viewer multislice CT. The coracoid tunnel diameter was measured at the widest tunnel diameter in the axial cuts (Fig. 1a), and the ratio to the widest coracoid horizontal diameter (Fig. 1b) was calculated, as this ratio gives a better indication for the risk of coracoid fracture. These radiological measures (CT and radiographs) were independently measured by two surgeons twice to ensure reliability testing. In the case of a minor difference (≤ 2 mm) between investigators, the mean of the two measurements was calculated. Retesting was performed when a major difference was encountered (> 2 mm). The Constant Shoulder Score, Oxford Shoulder Score and Nottingham Clavicle Score were used as outcome measures of ACJ function and disability [1, 5]. A visual analog scale (VAS score) was used to score pain at the distal end of the clavicle, which reflects ACJ arthritis [17]. A clinical failure was set as a shoulder Constant score < 85 points [8].

### Statistical methods

The sample size was calculated using the G-power program (version 3.1.9.7). The effect size was 0.71 and the power of the test was 80%. The minimum sample size needed for this study was 14 patients, and 9 extra patients were included to avoid any missing data during follow-up. Data were coded and entered using SPSS version 25 (IBM, USA). The data are presented as the mean and standard deviation (SD) or as the number and percentage. Comparisons between quantitative variables were performed using the Wilcoxon signed rank test or paired *t* test. Correlations between quantitative variables were performed using the Spearman correlation coefficient. The confidence interval was set to 95%, and the margin of error accepted was set to 5%. *P* values less than 0.05 were considered statistically significant.

## Results

Twenty-three patients with grade III-V ACJ dislocation eligible for arthroscopic fixation using the TightRope System (Arthrex Inc, USA Naples) were included in the study and followed up for 12 months.

The functional clinical scores at 12 months postoperatively are shown in Table 1. There were 3 cases of clinical failure (4.3%). The coracoid tunnel diameter to horizontal coracoid diameter ratio increased from 22.8 ± 3.7% immediately postoperatively to 38.5 ± 5.5% at 12 months by 15.7% (*p* < 0.001) (Table 2). This demonstrates significant coracoid tunnel widening. However, no correlation was found between the degree of injury according to the Rockwood classification and tunnel widening (correlation coefficient 0.263).

**Table 1:** Functional clinical scores at 12 months postoperative

	Mean	Standard deviation	Median	Minimum	Maximum
VAS at 12 m	1.4	1.5	1	0	5
Constant shoulder score at 12 months postoperative	87.3	7	88	70	96
Oxford shoulder score at 12 months postoperative	43	2.2	44	39	46
Nottingham clavicle score at 12 months postoperative	93.6	4.8	94	82	100

**Table 2:** Coracoid tunnel dilatation and CC distance at immediate postoperative and at 12 months postoperative

	Mean	Standard deviation	Median	Minimum	Maximum	*P* value
Coracoid tunnel diameter to whole coracoid diameter at immediate postoperative%	22.8	3.7	22.3	16.5	29.5	< 0.001
Coracoid tunnel diameter to whole coracoid diameter at 12 months postoperative%	38.5	5.5	38.6	30.9	49.3	
Coraco-clavicular distance at immediate postoperative	10.8	1.7	11	8	13	< 0.001
Coraco-Clavicular distance at 12 months postoperative	11.8	2.5	12	8	16	

The CC distance representing reduction showed an increase from a mean of 10.8 ± 1.7 mm immediately postoperatively to a mean of 11.8 ± 2.5 at 12 months (*p* < 0.001); this signifies loss of reduction over time (Table 2). Despite loss of reduction, none of the patients were classified as radiological failure.

Regarding the correlation between functional scores and CC distance, as illustrated in Table 3, there was no correlation between functional clinical scores of the patient and loss of reduction. There was also no correlation between coracoid tunnel widening and an increase in the CC distance, which represented a loss of reduction at the final follow-up (Table 3).

**Table 3:** Coraco-clavicluar (CC) distance correlation with functional scores and coracoid tunnel dilatation

	Coraco-clavicular [12 months post-op] (mm)
*Constant shoulder score at 12 months Post-operative*	
Correlation coefficient	– 0.1
*P* value	0.6
*N*	23
*Oxford shoulder score at 12 months Post-operative*	
Correlation coefficient	– 0.2
*P *value	0.3
*N*	23
*Nottingham clavicle score at 12 months * *Post-operative*	
Correlation coefficient	– 0.1
*P* value	0.8
*N*	23
*Coracoid tunnel dilatation*	
Correlation coefficient	0.2
*P* value	0.4
*N*	23

## Discussion

The most important finding in the present study did not fully prove the initial hypothesis. There was significant coracoid tunnel widening and significant increase in CC distance after arthroscopic ACJ fixation using TightRope system. However, there was no correlation between coracoid tunnel widening and loss of reduction and this did not significantly affect functional outcomes, and none of the patients were considered radiological failure. Although loss of reduction did not significantly affect functional outcomes, there were 3 cases who were classified as clinical failure with a Constant score of < 85 at final follow-up.

Several open and arthroscopic or arthroscopic-assisted techniques have been described for the treatment of AC joint injuries, each with its advantages and limitations. Restoring the anatomy and stability is the aim of surgery and is key to its success and good functional outcomes. Residual chronic pain and loss of reduction are the most common complications post-ACJ surgery and a major cause of patient dissatisfaction [3].

Arthroscopic techniques have been gaining popularity using smaller wounds to minimize the hospital stay, postoperative pain, and adhesions, which enables quicker recovery. Such techniques allow adequate visualization of the coracoid undersurface and precise tunnel placement when required [6]. They also allow detection and management of associated intra-articular pathologies, which in one study have been estimated to have a prevalence of 42.8% [2]. Despite these advantages and good functional outcomes, radiological failure and loss of reduction are still concerns [14, 21, 29].

One of the most used arthroscopic techniques is using a double tight rope and fiber-wire or fiber-tape for fixation as a brace to allow healing of the ligaments. This technique depends on the drilling of a tunnel into the clavicle and the coracoid [23].

There is concern that tunnel drilling is associated with the risk of coracoid and/or clavicular fractures or tunnel widening. This is hypothesized to be a possible cause of loss of reduction. In a systematic review by Nelson et al. [20], the pooled results from 12 studies (after filtration) included a total of 221 patients (out of 246) who were followed up for an average of 20.4 months. The combined rate of fracture of the clavicle or coracoid was 5.3%, with 7 coracoid fractures and 1 fracture clavicle. The rate of reduction loss was 26.1% and ranged widely between studies. In the series of Kany et al., it was 6.5% [13], while Cook et al. reported a loss of reduction in 80% of their cases [9]. It is worth noting that most of the reduction losses occurred in patients with chronic ACJ injuries, not acute injuries. In this study, patients with acute ACJ injuries (within 3 weeks after injury) were included.

Thangaraju et al. have described clavicular tunnel dilatation and have quantified reduction on Zanca radiographs views after a mean follow-up of 6 months with only 13 patients [30]. This study relies on a more accurate objective tool: CT scan for assessment of coracoid tunnel widening, for which the sample size is larger and has a longer follow-up. To the authors' knowledge, no other clinical study has attempted to measure and quantify coracoid tunnel widening on CT scans and its association with loss of reduction. The theory of tunnel widening lies in the windshield-wiper and bungee effects of the graft. Tunnel widening was demonstrated in this study by an average of 15.7% at 12 months of follow-up. In their series, Thangaraju et al. did not find a correlation between *clavicular* tunnel widening and radiological failure. This is similar to this series, where a correlation was not found between *coracoid* tunnel widening and loss of reduction.

In another study by Berthold et al. [4], 24 patients who underwent CC ligament reconstruction using tendon allografts were evaluated and followed up for a mean of 37 months. Medial and lateral clavicular tunnels were drilled in the anatomical position of the CC ligament attachment. Radiographs were used to assess clavicular tunnel widening and demonstrated significant tunnel widening in both the medial and lateral clavicular tunnels. There was also a significant loss of reduction (increased CC distance over time); however, there was no correlation between the two factors, and they did not correlate with poor clinical outcomes. This is consistent with the findings of this study. Despite having a longer follow-up, the researchers obtained similar results to this study. Furthermore, despite reconstructing the ACJ joint capsule, which may confer better stability, there was a significant increase in CC distance from 6.2 mm to 10.1 mm between the early postoperative to the latest postoperative follow-up. However, no significant correlation between loss of reduction and medial or lateral tunnel widening was found.

In their study, Shin et al. reported a 33% loss of reduction. They proposed the use of double tunnels to improve the construct and confer greater stability [28]. However, the double tunnel itself is at risk of tunnel widening. However, Klabklay et al. in their study proposed that over-reduction of the ACJ minimizes loss of reduction and therefore confers better outcomes. In their technique, they described over-reduction by 50% of the sound side. It is however, challenging and difficult to achieve this [15].

Milewski et al. [19] studied the use of CC reconstruction using a tendon graft coupled with a cortical fixation button device, with and without using the coracoid tunnel. They reported fewer complications when the graft was simply looped under the coracoid with no coracoid tunnels drilled. With coracoid tunnel drilling, they had a 50 percent loss of reduction and a 100% failure rate. This study highlights the fact that merely drilling into the coracoid increases the risk of loss of reduction but does not correlate the position of the coracoid tunnel on CT with the loss of reduction. Loss of reduction and radiological failure after arthroscopic fixation of the AC joint by a tight rope may be explained by multiple factors. A biomechanical study showed that the size of the drill hole can affect outcomes; small drill holes have a lower risk of iatrogenic fracture [32], and for this reason, the ratio between the tunnel diameter and the coracoid diameter was used in this study. Another study analyzed risk factors for failure and complications of minimally invasive coracoclavicular ligament reconstruction using a flip button repair. The researchers noted that the site placement of the coracoid button is another important factor and that central positioning under the coracoid base is crucial to prevent failure [16].

Drilling of coracoid and clavicular tunnels prior to reduction of the ACJ may be a cause of later tunnel widening and subsequent loss of reduction. In this study, the first step was reduction of the ACJ followed by temporary stabilization with a k-wire, and then an image intensifier was used to check the quality of reduction. This ensures tunnel drilling in a reduced position and minimizes the possibility of tunnels not being in line and posing a risk of tunnel widening.

Zhang et al. reported a 25% loss of reduction in their series with lower outcome scores. They explained this with 2 possible reasons: cutting off the fiber-wire against the clavicular cortex and eccentric anterior drilling of the clavicular tunnel [33].

Implant migration is a recognized possible cause of radiological loss of reduction and possible failure. Using the TightRope or EndoButton technique, the most reported complication was hardware migration into the clavicle and/or coracoid. This rate reached 89% in the study of Scheibel et al. However, Scheibel et al. found no correlation between implant migration and loss of reduction or any difference regarding the CC distance on the contralateral side [27]. A similar study by Venjakob et al. reported no further migration of the hardware into the clavicular tunnels at the final follow-up of 58 months compared to the 24-month images. This suggests that the initial loss of reduction does not continue, explaining the good functional outcomes despite radiological failure [31].

This study is the first clinical study to quantify *coracoid tunnel widening* using CT. Furthermore, this is the first in vivo study to draw attention to the possible cause of failure and attempt to correlate it to coracoid tunnel widening. An in vitro study by Dalos et al. used micro-CT analysis to assess coracoid and clavicular tunnel widening and compared them across three different repair systems [10]. They reported tunnel widening at 5 different tunnel cross sections and found that tunnel widening mainly occurs at the inferior part of the clavicular tunnel followed by the superior part of the coracoid tunnel. However, this reflects only mechanical factors as a cause of tunnel widening and does not take into consideration biological factors that might be involved in the process. In this study, tunnel widening was assessed from the widest axial CT cut. It would be very useful to enlarge the measurements made to different parts of the tunnel using micro-CT.

This study has some limitations. The study did not compare tunnel widening with different methods and techniques of fixation, which rely on different drill hole diameters and different suture materials. Recent devices use smaller drill hole diameters, and knowing that the tunnel size affects tunnel widening, in this study, a ratio between tunnel size and the coracoid base diameter was relied on. It is therefore necessary to study tunnel widening, its correlation with loss of reduction and its effect on functional outcomes across different techniques of fixation. In this study, only one measurement of the tunnel diameter was performed, precisely, the widest part. It would be valuable to perform measurements at different cross sections of the tunnel to analyze which portion of the tunnel undergoes the greatest amount of widening. This study highlights the occurrence of coracoid tunnel dilatation after arthroscopic AC stabilization using TightRope system. To minimize the coracoid tunnel widening consequences, it is recommended to use a smaller diameter drill bit and tunnel. It is also recommended to use a single coracoid tunnel rather than double tunnels and to make this tunnel as central as possible in the coracoid.

## Conclusion

The main findings of this study did not fully prove the initial hypothesis. Both coracoid tunnel widening and radiological loss of reduction significantly occur after arthroscopic fixation of acute ACJ dislocation with the TightRope system. However, they do not correlate with the functional scores of the patient. Furthermore, no significant correlation is established between coracoid tunnel dilatation and loss of reduction.

## Data Availability

Data available on request from the authors.
